# Anchoring Pt Single Atoms on Te Nanowires for Plasmon‐Enhanced Dehydrogenation of Formic Acid at Room Temperature

**DOI:** 10.1002/advs.201900006

**Published:** 2019-04-17

**Authors:** Lei Han, Leijie Zhang, Hong Wu, Hualu Zu, Peixin Cui, Jiasheng Guo, Ruihan Guo, Jian Ye, Junfa Zhu, Xusheng Zheng, Liuqing Yang, Yici Zhong, Shuquan Liang, Liangbing Wang

**Affiliations:** ^1^ School of Materials Science and Engineering Key Laboratory of Nonferrous Metal Materials Science and Engineering Ministry of Education Central South University Changsha Hunan 410083 P. R. China; ^2^ National Synchrotron Radiation Laboratory University of Science and Technology of China Hefei Anhui 230029 P. R. China; ^3^ Key Laboratory of Soil Environment and Pollution Remediation Institute of Soil Science The Chinese Academy of Sciences Nanjing 210008 P. R. China

**Keywords:** dehydrogenation of formic acid, plasmonic catalysis, platinum, single atoms, tellurium

## Abstract

Formic acid (HCOOH), as a promising hydrogen carrier, is renewable, safe, and nontoxic. However, the catalytic dehydrogenation of HCOOH is typically conducted at elevated temperature. Here, HCOOH decomposition is successfully achieved for hydrogen production on the developed Pt single atoms modified Te nanowires with the Pt mass loading of 1.1% (1.1%Pt/Te) at room temperature via a plasmon‐enhanced catalytic process. Impressively, 1.1%Pt/Te delivers 100% selectivity for hydrogen and the highest turnover frequency number of 3070 h^−1^ at 25 °C, which is significantly higher than that of Pt single atoms and Pt nanoclusters coloaded Te nanowires, Pt nanocrystals decorated Te nanowires, and commercial Pt/C. A plasmonic hot‐electron driven mechanism rather than photothermal effect domains the enhancement of catalytic activity for 1.1%Pt/Te under light. The transformation of HCOO* to CO_2_ *^δ^* ^−^* on Pt atoms is proved to be the rate‐determining step by further mechanistic studies. 1.1%Pt/Te exhibits tremendous catalytic activity toward the decomposition of HCOOH owing to its plasmonic hot‐electron driven mechanism, which efficiently stimulates the rate‐determining step. In addition, hot electrons generated by the Te atoms nearby Pt single atoms are regarded to directly inject into the reactants adsorbed and activated on Pt single atoms.

Formic acid (HCOOH) is a renewable, safe, and nontoxic hydrogen storage chemical that contains 4.4 wt% hydrogen, and thus attracts extensive attention.^1^, ^2^, ^3^, ^4^, ^5^, ^6^, ^7^, ^8^, ^9^, ^10^, ^11^ Noble metal based catalysts such as Ir complexes, Ru‐biaryl sulfonated phosphines, bimetallic Pd/M (M = late transition fcc metal), PdAg nanoparticles, and PtCu single‐atom alloys have been serving as efficient catalysts for the dehydrogenation of HCOOH.^2^, ^3^, ^4^, ^5^, ^6^ Such catalysts suffered from the elevated temperature (60–150 °C) to enhance their performance, which became troublesome barriers to their usages in a wider range. Recently, a number of research groups have looked deep into boosting the catalytic activity at room temperature instead of harsh reaction conditions. Modulating the metal–support interaction is a feasible approach to construct efficient supported catalysts. For instance, Bulut et al. reported PdAg alloy and MnOx nanoparticles coloaded TiO_2_ catalysts with remarkable activity at room temperature.^7^ Besides, NiPd ultrafine particles supported on NH_2_‐functionalized and N‐doped graphene oxide were capable of catalyzing HCOOH selective dehydrogenation at 25 °C.^8^ In addition, modification of surface electronic properties was proved to be another promising method to enhance catalyst activity at room temperature. For instance, by coupling with pyridinic nitrogen–doped carbon to modulate the surface electronic properties, Pd nanoparticles achieved a turnover frequency (TOF) of 5530 h^−1^ at 25 °C.^9^ Wang et al. constructed a trimetallic CoAuPd/C catalyst in special surface electronic state with the final conversion reaching 91%.^10^ Moreover, Jiang et al. incorporated B atoms into Pd–Pd interlattice spaces to optimize the electronic structure of the catalyst, which exhibited excellent activity at room temperature.^11^ Although great progress has been achieved, highly efficient catalysts that can operate at room temperature for the dehydrogenation of HCOOH are still urgently desired.

Single‐atom catalysts are a class of heterogenous catalysts that contain isolated metal atoms dispersed singly on the support.^12^ Single‐atom catalysts exhibit remarkable catalytic activity and selectivity toward various catalytic reactions due to their high atomic utilization, low coordination environment, and strong metal–support interaction.^13^ Plasmonic catalysis has gained increasing interests recently.^14^ Catalytic process is driven by solar energy harvested by plasmonic metals (e.g., Au, Ag, Cu) via the localized surface plasmon resonance (LSPR) in plasmonic catalysis.^15^ Therefore, introducing plasmonic effect into single‐atom catalysis exhibits tremendous potential in simultaneously moderating reaction temperature as well as improving catalytic activity and selectivity.

Herein, we report a strategy to construct Pt single atoms modified Te nanowires (Te NWs) with the Pt mass loading of 1.1% (denoted as 1.1%Pt/Te) to achieve significant activity and selectivity for the dehydrogenation of HCOOH at 25 °C via plasmonic catalysis. By simply increasing Pt mass loading to 4.6% and 32.0%, Pt single atoms and Pt nanoclusters coloaded Te nanowires (denoted as 4.6%Pt/Te) and Pt nanocrystals supported Te nanowires (denoted as 32.0%Pt/Te) were obtained, respectively. During the catalytic tests, 1.1%Pt/Te achieved the 100% selectivity for H_2_ as well as the highest TOF number of 3070 h^−1^ within the obtained 1.1%Pt/Te, 4.6%Pt/Te, 32.0%Pt/Te, and commercial Pt/C under illumination at 25 °C. Plasmonic hot‐electron driven mechanism rather than photothermal effect played the leading role in the enhancement of catalytic activity for 1.1%Pt/Te under light. Further mechanistic studies revealed that the HCOO*‐to‐CO_2_ *^δ^* ^−^* conversion on Pt atoms was the rate‐determining step. 1.1%Pt/Te exhibited remarkably enhanced catalytic activity toward the decomposition of HCOOH because of its plasmonic hot‐electron driven mechanism under light, which efficiently stimulated the rate‐determining step. In addition, hot electrons generated by the coordinating Te atoms were regarded to directly inject into reactants adsorbed and activated on Pt single atoms.

To begin with, Te nanowires were synthesized with the diameter of ≈4–7 nm according to the method being reported previously (Figure S1, Supporting Information).^16^ Then 1.1%Pt/Te single‐atom catalysts were prepared by simply injecting H_2_PtCl_6_ aqueous solution (15 µL, 100 × 10^−3^ m) into ethylene glycol solution containing Te NWs, followed by stirring at 60 °C for 13 h. The Pt mass loading on Te NWs was determined to be 1.1% by the inductively coupled plasma atomic emission spectroscopy (ICP‐AES). **Figure** **1**A shows a high‐angle annular dark‐field scanning transmission electron microscopy (HAADF‐STEM) image of the as‐obtained 1.1%Pt/Te. Isolated Pt atoms were observed as brighter spots and marked with yellow circles, indicating the uniform dispersion on the surface of Te NWs. In addition, as shown in Figure S2 in the Supporting Information, the X‐ray diffraction (XRD) pattern of 1.1%Pt/Te could be indexed to Te (PDF No. 86‐2268). The procedures for the synthesis of 4.6%Pt/Te and 32.0%Pt/Te were similar to that for 1.1%Pt/Te, except for changing the amount of the H_2_PtCl_6_ aqueous solution from 15 µL to 75 and 360 µL, respectively. ICP‐AES result determined that the mass loading of Pt for 4.6%Pt/Te and 32.0%Pt/Te was 4.6% and 32.0%, respectively. As for 4.6%Pt/Te shown in Figure S3 in the Supporting Information, Pt single atoms could also be clearly observed and were marked with yellow circles. As shown in Figures S3 and S4 in the Supporting Information, Pt nanocrystals were in an average size of ≈4 nm for 32.0%Pt/Te. The interplanar distance of Pt nanocrystals was measured to be 2.2 Å, corresponding to the {111} planes of platinum. For later comparison, commercial Pt/C was directly purchased, with the size of Pt nanoparticles being 4–5 nm (Figure S5, Supporting Information). The X‐ray absorption near‐edge spectroscopy (XANES) and the extended X‐ray absorption fine structure (EXAFS) profiles were recorded to determine the electronic structure and the coordination of Pt atoms in 1.1%Pt/Te, 4.6%Pt/Te, and 32.0%Pt/Te, respectively. As shown in Pt L_3_‐edge XANES profiles (Figure 1B), the white line intensity for 1.1%Pt/Te, 4.6%Pt/Te, and 32.0%Pt/Te was significantly weaker than that for PtO_2_ but basically similar to that of Pt foil. Therefore, Pt species in 1.1%Pt/Te, 4.6%Pt/Te, and 32.0%Pt/Te were mainly in the metallic state. As a comparison, the white line intensity for Pt/C was between that of Pt foil and PtO_2_. Hence, Pt species in Pt/C was partly oxidized. As shown by the EXAFS spectrum in R space (Figure 1C), the coordination number (CN) for the Pt–Te shell in 1.1%Pt/Te was 4.5, without Pt–Pt coordination (Table S1, Supporting Information), revealing that Pt atoms were atomically dispersed throughout the whole Te NWs. For 4.6%Pt/Te, the CNs were determined to be 4.1 and 1.7 for the Pt–Te shell and the Pt–Pt shell, respectively, demonstrating the existence of both Pt single atoms and Pt nanoclusters on Te NWs. As for 32.0%Pt/Te, the CNs for the Pt–Pt shell and the Pt–Te shell were calculated to be 7.7 and 0.8, respectively, proving the formation of Pt nanocrystals on Te NWs. The wavelet transform (WT) analysis of Pt foil, 1.1%Pt/Te, 4.6%Pt/Te, and 32.0%Pt/Te in Pt L_3_‐edge were provided in Figure 1D–G. The location of the intensity maxima in 1.1%Pt/Te exhibited a left‐shift and a down‐shift in the wave vector *k* and the bond length *R* relative to Pt foil, respectively, indicating a lower atomic number and a shorter bond length, which could be ascribed to Te atoms. Also, the scattering peak at about (12.5 Å^−1^, 2.6 Å) for Pt atoms was disappeared, implying the single‐atom form for Pt atoms in 1.1%Pt/Te. As for 4.6%Pt/Te, the scattering peak at about (10.7 Å^−1^, 2.4 Å) was ascribed to both Pt–Te and Pt–Pt coordination. In comparison, the location of the peak in 32.0%Pt/Te was quite analogous to that of Pt foil, suggesting the aggregated form for Pt atoms in 32.0%Pt/Te. The EXAFS results were highly consistent with the HAADF‐STEM images. Figure 1H illustrates the ultraviolet–visible spectroscopy (UV–vis) of Te NWs, 1.1%Pt/Te, 4.6%Pt/Te, and 32.0%Pt/Te. Te NWs, 1.1%Pt/Te, and 4.6%Pt/Te possessed two broad absorption bands located at ≈285 and ≈640 nm, which were due to an allowed direct transition (3–6 eV) and a forbidden transition (0–3 eV) triggered by the LSPR of metal Te, respectively.^17^ In contrast, the absorption band of 32.0%Pt/Te was mainly located in the ultraviolet region.

**Figure 1:**
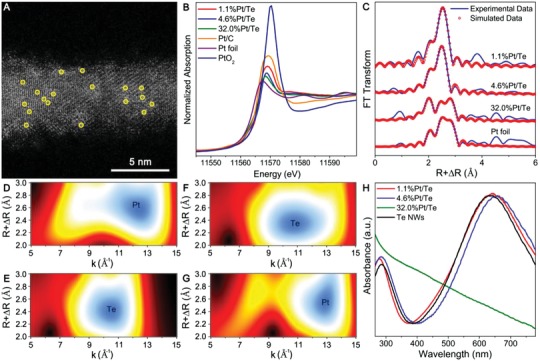
A) HAADF‐STEM image of 1.1%Pt/Te. B) Pt L_3_‐edge XANES spectra for 1.1%Pt/Te, 4.6%Pt/Te, 32.0%Pt/Te, and Pt/C. Pt foil and PtO_2_ were used as references. C) Pt L_3_‐edge EXAFS spectra in R space for 1.1%Pt/Te, 4.6%Pt/Te, 32.0%Pt/Te, and Pt foil. D–G) The wavelet transform in Pt L_3_‐edge for Pt foil, 1.1%Pt/Te, 4.6%Pt/Te, and 32.0%Pt/Te. H) UV–vis spectra of 1.1%Pt/Te, 4.6%Pt/Te, 32.0%Pt/Te, and Te NWs.

The catalytic properties of the as‐obtained 1.1%Pt/Te, 4.6%Pt/Te, 32.0%Pt/Te, and Pt/C for the dehydrogenation of HCOOH were evaluated in a home‐built catalytic system. Each reaction was catalyzed by the corresponding amount of catalyst (90.9 mg of 1.1%Pt/Te, 21.7 mg of 4.6%Pt/Te, and 3.1 mg of 32.0%Pt/Te) determined by ICP‐AES to ensure that the amount of Pt element for each catalyst was 1 mg. The light used in all experiments was projected by Xenon lamp of the same intensity (250 mW cm^−2^). Blank tests were conducted at 25 °C without any catalyst, in which almost no product was observed, regardless of the presence or absence of light. Then the reaction was tested at 25 °C with Te NWs (20.7 mg) added in a three‐neck flask containing 2 mL of HCOOH (1 m) and 10 mL of deionized water, where only 7.8 and 2.5 mL of gas was produced in case of lighting and no lighting, respectively (**Figure** **2**A). Thus, Te NWs had poor catalytic activity for the dehydrogenation of HCOOH. As for 1.1%Pt/Te, 4.6%Pt/Te, 32.0%Pt/Te, and Pt/C, less than 5 mL of gas was released after 40 min at 25 °C without light. In contrast, when the reaction was conducted under light, all of the above Pt‐based catalysts exhibited enhanced catalytic activity. Commercial Pt/C (20 mg, 5% mass loading) was tested as a reference, where 18 mL of gas was yielded after 40 min. 32.0%Pt/Te and 4.6%Pt/Te performed better than Pt/C, with 28 and 62 mL of gas produced. Under the same reaction condition, 1.1%Pt/Te exhibited a remarkably higher catalytic activity relative to Pt/C, with 89 mL of gas formed. In order to determine the composition of the obtained gas, we conducted gas chromatograph (GC) tests. According to the GC results, the gas released from the decomposition of HCOOH catalyzed by 1.1%Pt/Te, 4.6%Pt/Te, 32.0%Pt/Te, and Pt/C all consisted of H_2_ and CO_2_, which can be explained by Equation (1)

(1) $$\mathrm{HCOOH}\to H_{2}+{\mathrm{CO}}_{2}$$

**Figure 2:**
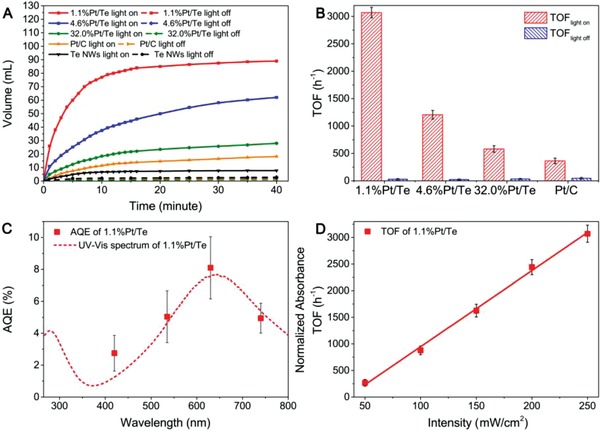
A) Plots of the volume of gas released from HCOOH decomposition catalyzed by different catalysts versus time at 0.1 MPa, 25 °C with light on/off. B) Comparison of TOF_light on_ and TOF_light off_ of different catalysts. C) Correlation between AQEs of 1.1%Pt/Te with light wavelength. D) Correlation between TOFs of 1.1%Pt/Te with light intensity.

According to Equation (1), catalyzed by 1.1%Pt/Te under light at 25 °C, more than 92% of HCOOH was converted to H_2_ and CO_2_ within 40 min. To quantitatively evaluate the catalytic activity, turnover frequency numbers based on Pt atoms within the initial 5 min were calculated. As shown in Figure 2B, the TOFs of 1.1%Pt/Te, 4.6%Pt/Te, 32.0%Pt/Te, and Pt/C under light were calculated to be 3070, 1205, 580, and 363 h^−1^, respectively. Thus, 1.1%Pt/Te was determined to be the optimum among all the as‐obtained catalysts with the TOF number being 2.5, 5.4, and 8.5 times higher than that of 4.6%Pt/Te, 32.0%Pt/Te, and Pt/C, respectively. Representative works on the dehydrogenation of HCOOH over past few years were listed in Table S2 in the Supporting Information. 1.1%Pt/Te was among the best with the TOF reaching 3070 h^−1^ at room temperature. In addition, the stability of 1.1%Pt/Te was also tested by recycling the catalyst (Figure S6, Supporting Information). After five rounds, almost 97% of the original activity was preserved, indicating the high stability of 1.1%Pt/Te. The HAADF‐STEM image of reused 1.1%Pt/Te showed that the Pt single‐atom structure was retained without aggregation (Figure S7, Supporting Information). Moreover, Pt 4f peaks at 71.3 and 74.5 eV aligned to the metallic state of Pt in the X‐ray photoelectron spectroscopy (XPS) spectrum remained unchanged after the catalytic reaction, further revealing the stability of 1.1%Pt/Te.

To further investigate the function of illumination, we tested the dehydrogenation of HCOOH catalyzed by 1.1%Pt/Te, 4.6%Pt/Te, and 32.0%Pt/Te under different light wavelength. Catalytic tests for 1.1%Pt/Te, 4.6%Pt/Te, and 32.0%Pt/Te under different light wavelength (420, 535, 630, 740, and ±20 nm) were implemented by using a monochromator, keeping the intensity the same (75 mW cm^−2^). The apparent quantum efficiency (AQE) for each catalyst was calculated based on the amount of H_2_ generated within the initial 5 min. 1.1%Pt/Te reached the highest AQE of 8% at ≈630 nm (Figure 2C). Interestingly, AQEs of 1.1%Pt/Te irradiated by light with different wavelength shared the same trend of the UV–vis spectrum. Therefore, the outstanding catalytic activity of 1.1%Pt/Te under illumination was proposed to be in relation with the LSPR of 1.1%Pt/Te. As for 4.6%Pt/Te, AQEs also well matched the UV–vis spectrum (Figure S8, Supporting Information). In comparison, AQEs showed deviation from the trend of the UV–vis spectrum for 32.0%Pt/Te.

Plasmonic electron‐driven mechanism and photothermal effect accompanied by plasmonic effect are the two widely investigated factors for the enhancement of catalytic activity under light. We conducted the dehydrogenation of HCOOH catalyzed by 1.1%Pt/Te, 4.6%Pt/Te, and 32.0%Pt/Te in oil bath at 25 °C. Temperatures of the reaction solution after light illumination (250 mW cm^−2^, 40 min) for 1.1%Pt/Te, 4.6%Pt/Te, and 32.0%Pt/Te were measured to be 39, 38, and 36 °C, respectively, confirming the existence of photothermal effect. It should be noted that the surface temperature of catalysts would be higher than that of the reaction solution. Hence, we directly conducted the reaction at elevated temperature (80 °C) to further investigate the role of photothermal effect in stimulating the chemical reaction. TOFs of 1.1%Pt/Te, 4.6%Pt/Te, and 32.0%Pt/Te at 80 °C without light were calculated to be 1387, 799, and 613 h^−1^, respectively (Figure S9, Supporting Information). Even at a high temperature of 80 °C, the TOF for 1.1%Pt/Te without light only came to 45% of that at 25 °C under light. As for 4.6%Pt/Te and 32.0%Pt/Te, TOFs at 80 °C without light reached 66% and 105% of those at 25 °C under light, respectively. Moreover, we performed catalytic tests under different light intensity for 1.1%Pt/Te, 4.6%Pt/Te, and 32.0%Pt/Te. We modulated the working current of Xeon lamp to project white light with different intensity ranging from 50 to 250 mW cm^−2^. Figure 2D showed that there was a linear dependence of TOFs on the light intensity for 1.1%Pt/Te, which is a characteristic of the plasmonic electron‐driven chemical reaction.^18^ In comparison, TOFs for 4.6%Pt/Te presented a trend of quasi‐exponential growth with the increase of the intensity, which became more apparent for 32.0%Pt/Te (Figure S8, Supporting Information). In principle, an exponential relationship between the reaction rate and the laser intensity is a signature of the photothermal effect driven catalytic process.^19^ Therefore, with the gradual increase of Pt mass loading from 1.1% to 32.0%, photothermal effect acts an increasing part in the enhancement of catalytic activity toward the dehydrogenation of HCOOH. As for 1.1%Pt/Te, plasmonic electron‐driven mechanism rather than photothermal effect plays the major role in boosting the catalytic activity.

To investigate the interaction between reactants and 1.1%Pt/Te, 4.6%Pt/Te, and 32.0%Pt/Te, we conducted in situ diffuse reflectance infrared Fourier transform (DRIFT) measurements. In order to simulate the reaction conditions, 1.1%Pt/Te, 4.6%Pt/Te, and 32.0%Pt/Te were exposed to HCOOH at 25 °C with/without light for 10 min, followed by measurements of in situ DRIFT (**Figure** **3**A). Without light, two sets of frequencies were observed for 1.1%Pt/Te. One set of frequencies at 2940, 1746, and 1216 cm^−1^ were assigned to the stretching vibration of C—H, the stretching vibration of C=O, and the stretching vibration of C—O in HCOOH* species on 1.1%Pt/Te, respectively. The other set of typical frequencies at 2870, 1400, and 1361 cm^−1^ corresponded to the stretching vibration of C—H, the bending vibration of C—H, and the symmetrical stretching vibration of COO for HCOO* species, respectively.^20^ When 1.1%Pt/Te was treated with HCOOH at 25 °C under light for 10 min, a new peak at 1558 cm^−1^ assigned to the stretching vibration for chemisorbed CO_2_ *^δ^* ^−^* intermediates emerged, together with peaks at 2334 and 2359 cm^−1^ corresponded to the physisorbed CO_2_* species.^21^ In addition, the intensity of the peaks for HCOOH* and HCOO* were attenuated. Thus, illumination was assumed to prompt the consumption of HCOO* and HCOOH* as well as the generation of CO_2_ *^δ^* ^−^* and CO_2_*, which manifested the decomposition of HCOOH to produce CO_2_. As for 4.6%Pt/Te and 32.0%Pt/Te, the similar evolutionary trend of peaks in the in situ DRIFT spectra had been observed after the treatment of HCOOH under light, except for the difference in the ratio of the peak area for (CO_2_* + CO_2_ *^δ^* ^−^*) species to that of (HCOOH* + HCOO*) species (Figures S10 and S11, Supporting Information). With the increase of Pt mass loading from 1.1% to 32.0%, the ratio of the peak area for (CO_2_* + CO_2_ *^δ^* ^−^*)/(HCOOH* + HCOO*) significantly decreased. Therefore, 1.1%Pt/Te had the highest efficiency in the HCOO*‐to‐CO_2_ *^δ^* ^−^* transform.

**Figure 3:**
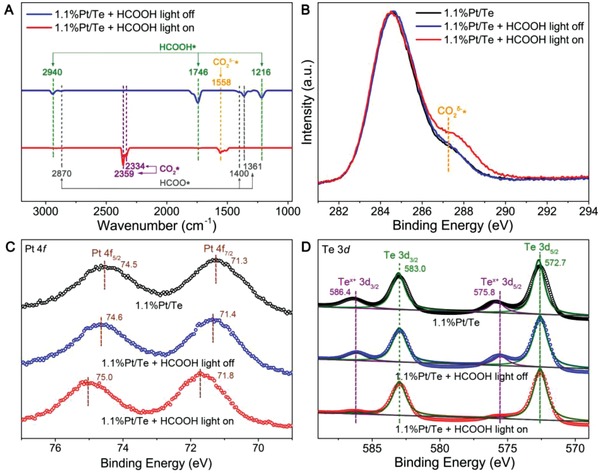
A) In situ DRIFT spectra of 1.1%Pt/Te after the treatment of HCOOH at 25 °C with/without light for 10 min. B–D) C 1s, Pt 4f, and Te 3d quasi in situ XPS spectra for 1.1%Pt/Te before and after the treatment of HCOOH at 25 °C with/without light for 10 min.

The interaction between reactants and 1.1%Pt/Te, 4.6%Pt/Te, and 32.0%Pt/Te was also revealed by quasi in situ XPS measurements. All quasi in situ XPS measurements of 1.1%Pt/Te, 4.6%Pt/Te, and 32.0%Pt/Te were conducted before and after the treatment of HCOOH at 25 °C with/without light for 10 min in a reaction cell attached to the photoemission end station at the National Synchrotron Radiation Laboratory (NSRL) in Hefei, China. It should be noted that the quasi in situ XPS measurements were carried out without exposure to light illumination. After the treatment of HCOOH in the absence of the light, the increase in the intensity of the peak at 287.3 eV for CO_2_ *^δ^* ^−^* in C 1s spectrum was negligible (Figure 3B). With the presence of light, the intensity of the peak for CO_2_ *^δ^* ^−^* was significantly enhanced. As such, illumination was the key to boost the transform of HCOO* to produce chemisorbed CO_2_ *^δ^* ^−^* intermediates via plasmonic hot‐electron driven mechanism for 1.1%Pt/Te. As shown in Figure 3C, the binding energy of Pt 4f after the treatment of HCOOH without light became only 0.1 eV higher than that before the treatment of HCOOH. In contrast, the increase in the binding energy of Pt 4f with light reached 0.5 eV. We speculated that electrons in Pt atoms partly transferred to the chemisorbed CO_2_ species to generate chemisorbed CO_2_ *^δ^* ^−^* intermediates, which led to the offset of Pt 4f peaks. Hence, Pt atoms were determined to be the active sites for the adsorption and the conversion of HCOO* to form chemisorbed CO_2_ *^δ^* ^−^* intermediates. Quasi in situ XPS spectra of Te 3d for 1.1%Pt/Te shown in Figure 3D exhibited predominant peaks at 583.0 and 572.7 eV, which were assigned to the metallic Te.^22^ Weaker peaks with higher binding energy were also found at 586.4 and 575.8 eV, illustrating that a part of Te atoms on the surface of 1.1%Pt/Te existed in oxidized Te*^x^* ^+^. The ratio of Te/Te*^x^* ^+^ in 1.1%Pt/Te after the treatment with HCOOH without light was almost the same as that before HCOOH treatment. Under illumination, the ratio of Te/Te*^x^* ^+^ increased remarkably. It was believed that the H_2_ released from the decomposition of HCOOH under light was adsorbed on the surface of 1.1%Pt/Te and reduced the Te*^x^* ^+^ species to metallic Te. As for 4.6%Pt/Te and 32.0%Pt/Te, we observed analogous variation tendency in peaks for C 1s, Pt 4f, and Te 3d quasi in situ XPS spectra (Figures S10 and S11, Supporting Information). It should be noted that the CO_2_ species produced from the decomposition of HCOOH presented three different forms, i.e., the physisorbed CO_2_*, chemisorbed CO_2_ *^δ^* ^−^* intermediates, and the released CO_2_. Only chemisorbed CO_2_ *^δ^* ^−^* intermediates could be detected in the quasi in situ XPS analysis chamber with a base pressure of <2 × 10^−10^ Torr. Among 1.1%Pt/Te, 4.6%Pt/Te, and 32.0%Pt/Te, 4.6%Pt/Te gained the highest CO_2_ *^δ^* ^−^* yield after the treatment of HCOOH under light for 10 min. As such, under light illumination for 10 min, the enhancement in the intensity of the peak for chemisorbed CO_2_ *^δ^* ^−^* intermediates in C 1s spectrum and the offset of peaks for Pt 4f were the most significant for 4.6%Pt/Te, which was highly consistent with in situ DRIFT measurements. Based on the above results, the reaction path for the dehydrogenation of HCOOH on 1.1%Pt/Te, 4.6%Pt/Te, and 32.0%Pt/Te was proposed as follows

(2) HCOOH*→HCOO*+H*

(3) $$\mathrm{HCOO*}\to {\mathrm{CO}}_{2}{}^{\delta -}*+\mathrm{H*}$$

(4) $${\mathrm{CO}}_{2}{}^{\delta -}*\to {\mathrm{CO}}_{2}$$

(5) $$\mathrm{2H*}\to H_{2}$$

The transform of HCOO* to CO_2_ *^δ^* ^−^* was believed to be the rate‐determining step, which was stimulated by light illumination. With the Pt mass loading increasing from 1.1% to 32.0%, the reaction rate of the HCOO*‐to‐CO_2_ *^δ^* ^−^* transform boosted by light was reduced, which led to declining catalytic activity toward the dehydrogenation of HCOOH. Considering that the light‐enhanced catalytic activity of 1.1%Pt/Te was originated from plasmonic hot‐electron driven mechanism while that of 32.0%Pt/Te was largely dependent on photothermal effect, we speculate that differences among the catalytic activity of 1.1%Pt/Te, 4.6%Pt/Te, and 32.0%Pt/Te under light were rooted from differences in these two mechanisms. Thus, relative to photothermal effect, plasmonic hot‐electron driven mechanism presents higher efficiency in catalyzing the decomposition of HCOOH. Therefore, 1.1%Pt/Te single‐atom catalyst exhibited tremendous catalytic activity in the dehydrogenation of HCOOH not only from its high atomic utilization, but also from its plasmonic hot‐electron driven mechanism upon light illumination.

Density functional theory (DFT) calculations were carried out to gain atomic‐level insight into how the hot electrons generated from the LSPR boosted the catalytic activity toward HCOOH decomposition for 1.1%Pt/Te. A model named as Pt‐S/Te was built for 1.1%Pt/Te by anchoring Pt single atoms on Te NWs (**Figure** **4**A). As shown in Figure 4B, the calculated projected density of states (PDOS) for Pt‐S/Te exhibited a gap at the Fermi level, indicating the semiconductor properties of 1.1%Pt/Te. The red line illustrated the independent contribution of Pt single atoms to the PDOS of Pt‐S/Te. Pt single atoms exhibited almost no PDOS near the Fermi level (±1 eV), which could be ascribed to the strong chemical bond between Pt single atoms and the coordinating Te atoms. In this case, it was almost impossible for hot electrons to inject into the band structure of Pt single atoms in the vicinity of the Fermi level.^23^ Considering Pt existed in the form of tiny single atom surrounded tightly by coordinating Te atoms, we proposed that hot electrons generated from nearby Te atoms directly injected to the reactants adsorbed on Pt single atoms to boost the conversion of HCOO* to CO_2_ *^δ^* ^−^* under light. As for 32.0%Pt/Te, it exhibited Pt nanoclusters loaded Te nanowires heterostructure. As such, there would be energy dissipation during the transfer of hot electrons from Te nanowires to Pt nanoclusters, which probably led to photothermal effect dominating the enhancement of catalytic activity.

**Figure 4:**
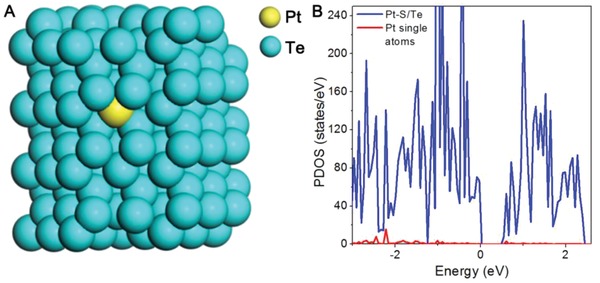
A,B) The optimized crystal structure and the calculated PDOS of Pt‐S/Te and Pt single atoms.

In conclusion, we report a facile synthesis of 1.1%Pt/Te which performed remarkable activity and selectivity toward HCOOH decomposition at 25 °C via plasmonic catalysis. During the dehydrogenation of HCOOH, the TOF of 1.1%Pt/Te reached the highest 3070 h^−1^ under light at room temperature, which was 2.5, 5.4, and 8.5 times as high as those of 4.6%Pt/Te, 32.0%Pt/Te, and Pt/C, respectively. Plasmonic hot‐electron driven mechanism rather than photothermal effect domains the enhancement of catalytic activity for 1.1%Pt/Te under light. Further mechanistic studies proved that the rate‐determining step of the dehydrogenation of HCOOH was the conversion of HCOO* to CO_2_ *^δ^* ^−^*. Plasmonic hot‐electron driven mechanism for 1.1%Pt/Te under light showed high efficiency in boosting the rate‐determining step, resulting in the enhancement of catalytic activity. In addition, hot electrons are regarded to directly inject into the reactants adsorbed and activated on Pt single atoms. This work not only develops a catalyst to achieve the reaction of HCOOH decomposition at room temperature, but also advances the understanding in specific reaction mechanism of the dehydrogenation of HCOOH via plasmonic catalysis.

## Conflict of Interest

The authors declare no conflict of interest.

## Supporting information

Supplementary
