# Supplementary material for: Anchoring Pt Single Atoms on Te Nanowires for Plasmon‐Enhanced Dehydrogenation of Formic Acid at Room Temperature
Source: Adv Sci (Weinh). 2019 Apr 17;6(12):1900006. doi: 10.1002/advs.201900006 (PMC6662073; doi:10.1002/advs.201900006)
Supplement: Supplementary file 1 — Supplementary [file ADVS-6-1900006-s001.pdf]

Copyright WILEY-VCH Verlag GmbH & Co. KGaA, 69469 Weinheim, Germany, 2019.

ADVANCED SCIENCE
Open Access

Supporting Information

for *Adv. Sci.,* DOI: 10.1002/advs.201900006

Anchoring Pt Single Atoms on Te Nanowires for Plasmon-Enhanced Dehydrogenation of Formic Acid at Room Temperature

Lei Han, Leijie Zhang, Hong Wu, Hualu Zu, Peixin Cui, Jiasheng Guo, Ruihan Guo, Jian Ye, Junfa Zhu, Xusheng Zheng, Liuqing Yang, Yici Zhong, Shuquan Liang,* and Liangbing Wang**

Experimental Section

Chemicals and materials. Polyvinylpyrrolidone (PVP, MW ~40,000), ammonia solution (NH_3, 25-28%), formic acid (HCOOH, 98%), ethylene glycol (EG, ≥99%), and chloroplatinic acid hexahydrate ($H_2PtCl_6 \cdot 6H_2O$, ≥99%) were obtained from Sinopharm Chemical Reagent Co., Ltd. Sodium tellurite (Na_2TeO_3, 97%) was purchased from Shanghai Macklin Biochemical Co., Ltd. Platinum on carbon (Pt/C, 5% mass loading) was obtained from Sigma-Aldrich. Hydrazine hydrate ($H_4N_2 \cdot H_2O$, 80%) was obtained from Xilong Scientific Co., Ltd. All solvents and chemicals were in analytical grade and used as received without further purification. All aqueous solutions were prepared using deionized water with a resistivity of 18.2 MΩ·cm.

Preparation of Te NWs. In a typical synthesis of Te nanowires (Te NWs),[S1] 2.000 g of PVP and 0.184 g of Na_2TeO_3 were dissolved in 70 mL of deionized water under vigorous magnetic stirring for 30 min at room temperature. After that, 6.7 mL of aqueous ammonia solution and 3.3 mL of hydrazine hydrate were added into the mixed solution, followed by stirring vigorously for another 10 min. Then the solution was transferred to a 100 mL Teflon-lined stainless steel autoclave and maintained at 180 ^{o}C for 3 h. After the autoclave was cooled down to room temperature naturally, the product was precipitated by acetone, washed three times with water and ethanol, and then re-dispersed into 30 mL of EG to form Te NWs solution (0.8 mg/mL, determined by ICP-AES).

Preparation of 1.1%Pt/Te, 4.6%Pt/Te, and 32.0%Pt/Te. For the synthesis of 1.1%Pt/Te**,** 15 μL of H_2PtCl_6 aqueous solution (100 mM) was added into 27 mL of EG containing Te NWs. After that, the formed solution was transferred into a pressure reaction vessel. Pure N_2 (1 bar) was bumped into the pressure reaction vessel to form an oxygen-excluded environment. Then the vessel was kept at 60 ^{o}C for 13 h under magnetic stirring at the speed of 240 rpm in an oil bath. Finally, the obtained product was precipitated by acetone, washed three times with water and ethanol, and then dried at 60 ^{o}C under vacuum. Further ICP-AES result determined that the mass loading of Pt for 1.1%Pt/Te was 1.1%. The synthetic process of 4.6%Pt/Te and 32.0%Pt/Te was similar to that of 1.1%Pt/Te, but the amount of H_2PtCl_6 aqueous

solution (100 mM) increased to 75 and 360 μL, respectively. ICP-AES result determined that the mass loading of Pt for 4.6%Pt/Te and 32.0%Pt/Te was 4.6% and 32.0%, respectively.

XAFS measurements for Pt element. The X-ray absorption fine structure (XAFS) spectra at Pt L_3-edge (E_0 = 11,564 eV) were performed at BL14W1 beam line of Shanghai Synchrotron Radiation Facility (SSRF) operated at 3.5 GeV under 'top-up' mode with a constant current of 220 mA. The XAFS data on 1.1%Pt/Te, 4.6%Pt/Te, and 32.0%Pt/Te samples were recorded under fluorescence mode with a Lytle detector. The energy was calibrated according to the absorption edge of pure Pt foil. Athena and Artemis codes were used to extract the data and fit the profiles. For the X-ray absorption near edge structure (XANES) part, the experimental absorption coefficients as a function of energies μ(E) were processed by background subtraction and normalization procedures. We refer to this process as 'normalized absorption'. For the extended X-ray absorption fine structure (EXAFS) part, the Fourier-transformed (FT) data in R space were analyzed by applying the 1st shell approximation or metallic Pt model for the Pt-Te or Pt-Pt shell, respectively. The passive electron factors, S_0^2, were determined by fitting the experimental Pt foil data and fixing the Pt-Pt coordination number (*CN*) to be 12, and then fixed for further analysis of the measured samples. The parameters describing the local structure environment including *CN*, bond distance (*R*) and Debye-Waller (*DW*) factor around the absorbed atoms were allowed to vary during the fit process. Wavelet transform (WT) analysis was employed using the Igor pro script developed by Funke et al.[S2] The Morlet wavelet was chosen as basis mother wavelet and the parameters (η =6, σ= 1) were used for a better resolution in the wave vector k.

Catalytic tests. Catalytic reactions were carried out in a 100 mL three-neck round bottom flask immersed into an oil bath. The volume of the gas generated during the catalytic reaction was monitored by a gas burette system. Xenon lamp (Perfectlight PLS-SXE300) was used to project light. According to ICP-AES results, 90.9 mg of 1.1%Pt/Te, 21.7 mg of 4.6%Pt/Te, 3.1 mg of 32.0%Pt/Te, and 20 mg of Pt/C (5% mass loading) were added into the three-neck flask containing 2 mL of

HCOOH (1M) and 10 mL of deionized water at 25 ^{o}C with/without light (250 mW/cm^{2}) illumination for 40 min, respectively. 20.7 mg of Te NWs were tested under the same reaction condition as a reference. After the catalytic reaction, the temperature of the reaction solution for each catalyst was measured by a thermocouple. The composition of released gas was determined by gas chromatogragh (Shimadzu GC-2014C). We modulated the working current of the Xenon lamp to project white light with different intensity (50 mW/cm^{2}~250mW/cm^{2}) to test 1.1%Pt/Te, 4.6%Pt/Te, and 32.0%Pt/Te under different light intensity. Catalytic tests for 1.1%Pt/Te, 4.6%Pt/Te, and 32.0%Pt/Te under light with different wavelength (420 nm, 535 nm, 630 nm, 740 nm, ±20 nm) were carried by using a monochromator, keeping the intensity the same (75 mW/cm^{2}). The irradiation area was measured to be 113 cm^{2}. The TOFs of 1.1%Pt/Te, 4.6%Pt/Te, 32.0%Pt/Te, and Pt/C were calculated based on Pt atoms within the initial 5 min. The AQE at specific wavelength (420 nm, 535 nm, 630 nm, 740 nm) for 1.1%Pt/Te, 4.6%Pt/Te, and 32.0%Pt/Te was determined by the following equation:

$$AQE=\frac{N_e}{N_p}=\frac{2\cdot N_H}{N_p}=\frac{2\cdot n_H\cdot N_A}{\frac{W\cdot A\cdot t}{h\cdot \nu}}\times 100\%$$

Where N_e and N_p represent the number of reacted electrons and incident photons. N_H and n_H are the number and the molar number of the generated H_2. W, A, and t are the intensity of the incident light, the irradiation area, and the time, respectively. ν represents the frequency of the incident light. N_A and h are the Avogadro's constant and the Planck constant.

***In situ* DRIFTS measurements.** *In situ* DRIFTS experiments were conducted in an elevated-pressure cell (Harrick DRK-4-BR4) with a Fourier transform infrared spectrometer (Bruker TENSOR II) with a wavenumber resolution of 4 cm^{-1} at 25 ^{o}C. After flowing with 1 bar of N_2 for 10 min at 25 ^{o}C, the background spectrum of sample was acquired. Then 1 bar of N_2 was allowed to bubble in HCOOH and flowed into the cell at 25 ^{o}C for 10 min with/without light for 1.1%Pt/Te, 4.6%Pt/Te, and 32.0%Pt/Te, followed by *in situ* DRIFT measurements.

***Quasi in situ* XPS measurements.** *Quasi in situ* XPS measurements were

performed at the photoemission end-station at beamline BL10B in the National Synchrotron Radiation Laboratory (NSRL) in Hefei, China. The beamline is connected to a bending magnet and covers photon energies from 100 to 1,000 eV with a resolving power ($E/\Delta E$) better than 1,000, and the photon flux was 1 $\times 10^{10}$ photons per s. The end-station is composed of four chambers: an analysis chamber, a preparation chamber, a load-lock chamber, and a high-pressure reactor. The analysis chamber, with a base pressure of $< 2 \times 10^{-10}$ torr, is connected to the beamline with a VG Scienta R3000 electron energy analyser and a twin anode X-ray source. The high-pressure reactor contains a reaction cell where the samples can be treated with different gases up to 20 bar and simultaneously heated up to 650 ^{o}C. After the sample treatment, the reactor can be pumped with pressure down to $< 10^{-8}$ torr for sample transfer. In the current work, 1.1%Pt/Te, 4.6%Pt/Te, and 32.0%Pt/Te were treated with HCOOH bubbled by 1 bar N_2 at 25 ^{o}C with/without light for 10 min in the high-pressure reactor and then transferred to the analysis chamber for *quasi in situ* XPS measurement without exposure to air.

DFT calculations. All the calculations were performed within spin-polarized DFT implemented in Vienna ab initio simulation package (VASP)[S3,S4] with the projector augmented wave (PAW)[S5] pseudopotentials for core−electrons. The generalized gradient approximation (GGA)[S6] with the Perdew-Burke-Ernzerhof (PBE) functional[S7] was adopted to treat the exchange-correlation interaction. A cut-off energy of 520 eV was applied for the plane wave expansion of valence electron wave functions. The unit lattice parameters of bulk Te were calculated to be a=4.41 and c=5.92 Å, which were close to the previous calculated values of a=4.45 and c=5.93 Å. [S8.S9] To model the Te NWs, a double-shelled Te NWs with 57 Te atoms per cell and a diameter of about 22 Å was constructed, the NWs is periodic in the *c* direction but were confined in the ab plane. The in-plane lattice parameter of 35 Å was set for the 1 × 1× 2 NWs supercell to avoid the interaction between the periodic images. A 1 × 1× 3 Monkhorst-Pack mesh *k* points in the irreducible Brillouin zone for geometric optimization and self-consistent calculations. One Te atom on the surface of the Te NWs was substituted by one Pt atom to build the

Pt-S/Te model for 1.1%Pt/Te. The empirical correction in Grimme's scheme (DFT-D3) approach[S10] was used to account for the van der Waals interactions.

Instrumentations. TEM and HAADF-STEM images were collected on a JEOL ARM-200F field-emission transmission electron microscope operating at 200 KV accelerating voltage. ICP-AES (Atomscan Advantage, Thermo Jarrell Ash, USA) was used to determine the concentration of Pt and Te. XRD pattern was recorded by using a Philips X'Pert Pro Super diffractometer with Cu-Kα radiation (λ=1.54178 Å). UV-Vis tests were conducted on a TU-1901 at room temperature.

References

[S1] H. W. Liang, S. Liu, Q. S. Wu, S. H. Yu, *Inorg. Chem.* **2009**, *48*, 4927.

[S2] H. Funke, A. Scheinost, M. J. P. R. B. Chukalina, *Phys. Rev. B* **2005**, *71*, 094110.

[S3] G. Kresse, J. Furthmüller, *Comp. Mater. Sci.* **1996**, *6*, 15.

[S4] G. Kresse, J. Furthmüller, *Phys. Rev. B* **1996**, *54*, 11169.

[S5] P. E. Blöchl, *Phys. Rev. B* **1994**, *50*, 17953.

[S6] J. P. Perdew, K. Burke, M. Ernzerhof, *Phys. Rev. Lett.* **1996**, *77*, 3865.

[S7] J. P. Perdew, M. Ernzerhof, K. Burke, *J. Chem. Phys.* **1996**, *105*, 9982.

[S8] P. Ghosh, M. U. Kahaly, U. V. Waghmare, *Phys. Rev. B* **2007**, *75*, 245437.

[S9] J. W. Liu, J. Xu, W. Hu, J. L. Yang, S. H. Yu, *ChemNanoMat* **2016**, *2*, 167.

[S10] S. Grimme, *J. Comput. Chem.* **2006**, *27*, 1787.

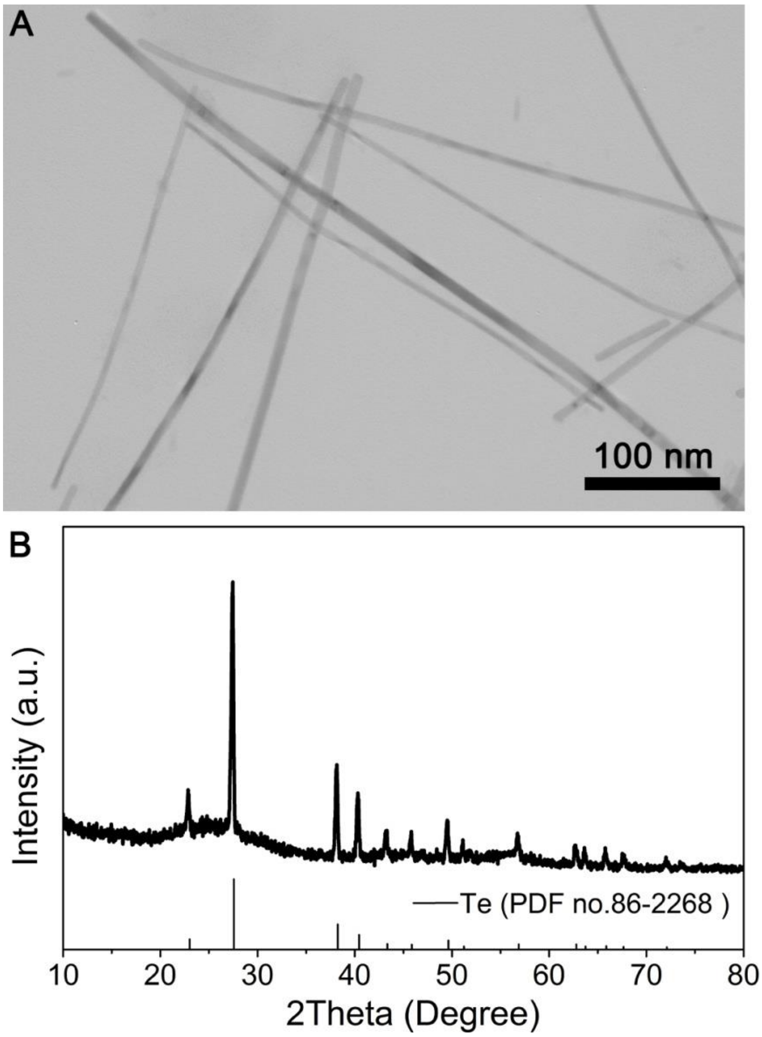

Figure S1. (A) TEM image and (B) XRD pattern of Te NWs.

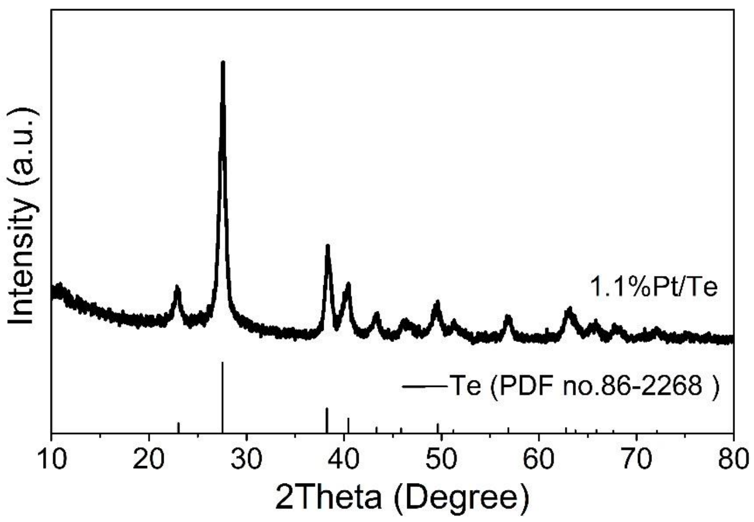

Figure S2. The XRD pattern of 1.1%Pt/Te.

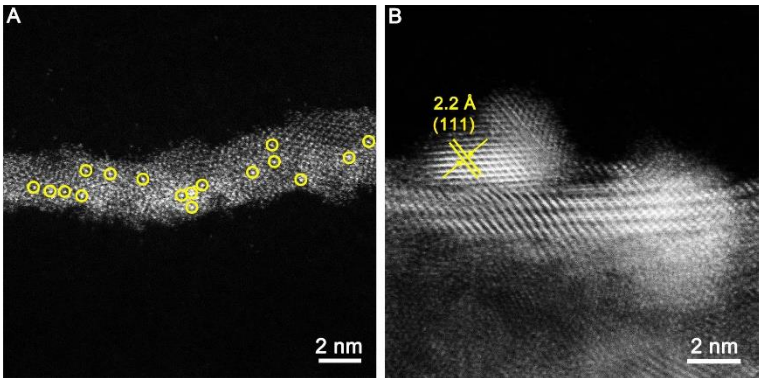

Figure S3. HAADF-STEM images of (A) 4.6%Pt/Te and (B) 32.0%Pt/Te.

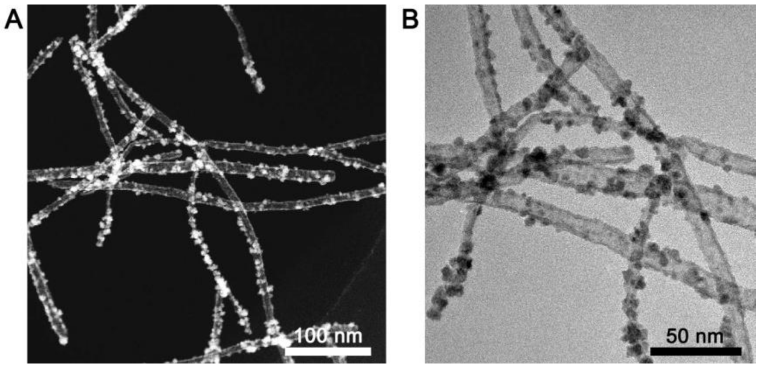

Figure S4. TEM images of 32.0%Pt/Te in (A) dark field and (B) light field.

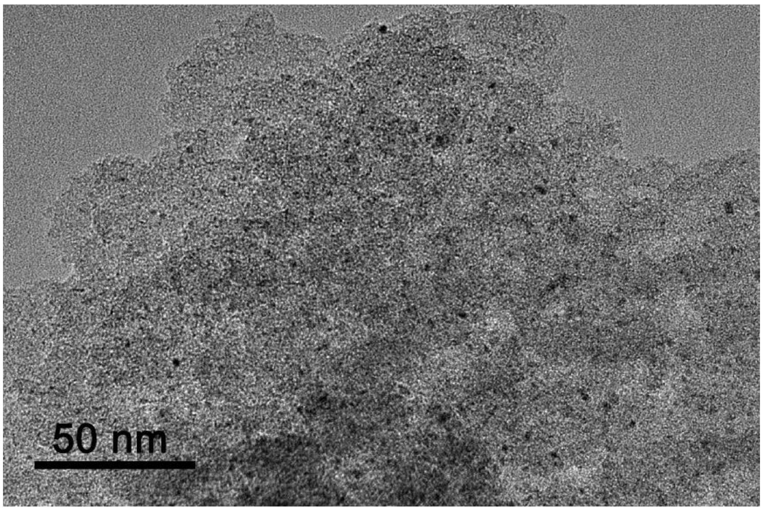

Figure S5. TEM image of the commercial Pt/C.

Table S1. EXAFS fitting results at Pt L_3-edge for 1.1%Pt/Te, 4.6%Pt/Te, and 32%Pt/Te.

Sample	Shell	*CN*	*R*(Å)	*D. W.*	ΔE_0 (eV)	*R* factor
Pt foil	Pt-Pt	12	2.76	0.0044	6.6	0.00068
1.1%Pt/Te	Pt-Te	4.5	2.65	0.0023	6.1	0.00153
4.6%Pt/Te	Pt-Te	4.1	2.61	0.0024	0.3	0.00094
	Pt-Pt	1.7	2.78	0.0043		
32.0%Pt/Te	Pt-Te	0.8	2.49	0.0007	4.3	0.00015
	Pt-Pt	7.7	2.72	0.0053		

CN, coordination number; *R*(Å), bond distance; *D. W.*, Debye-Waller factor; ΔE_0, the inner potential correction to account for the difference in the inner potential between the sample and the reference compound; *R* factor, goodness of fit. S_0^2 was set to be 0.794, according to the experimental EXAFS fit of Pt foil reference by fixing CN as the known crystallographic value.

Table S2. Catalytic performance for the dehydrogenation of formic acid by reported catalysts.

Catalyst	T (K)	TOF (h^{-1})	Ref.
$Ni_{0.4}Pd_{0.6}/NH_2$-N-rGO	298	954	*Adv. Mater.* **2018**, *30*, 1703038
$Pd/CN_{0.25}$	298	5530	*Angew. Chem. Int. Ed.* **2016**, *55*, 11849
Pd/S-1-in-K	323	3027	*J. Am. Chem. Soc.* **2016**, *138*, 7484
Au@Schiff-SiO_2	323	4368	*Energy Environ. Sci.* **2015**, *8*, 3204
AgPd	323	2739	*J. Am. Chem. Soc.* **2015**, *137*, 106
Pd/MSC-30	298	750	*Energy Environ. Sci.* **2015**, *8*, 478
Pd-B/C	298	1184	*J. Am. Chem. Soc.* **2014**, *136*, 4861
$Ag_{42}Pd_{58}$	323	382	*Angew. Chem. Int. Ed.* **2013**, *52*, 3681
$Co_{0.30}Au_{0.35}Pd_{0.35}/C$	298	80	*Angew. Chem. Int. Ed.* **2013**, *52*, 4406

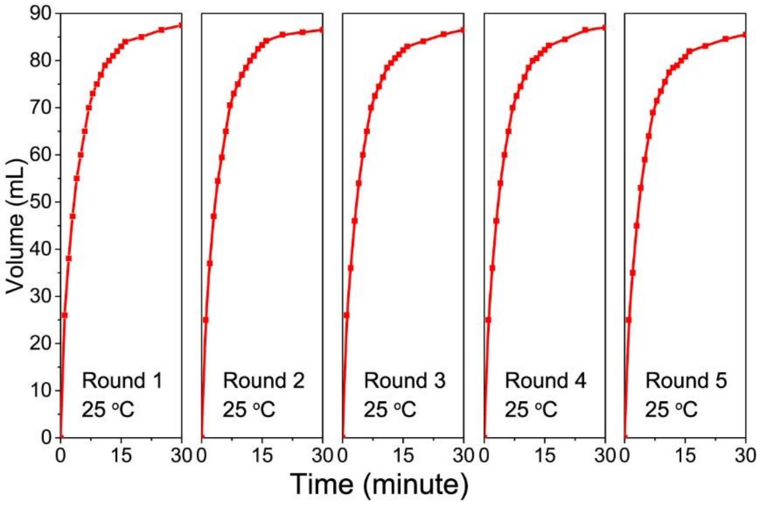

Figure S6. Catalytic performance of 1.1%Pt/Te after successive rounds of reaction.

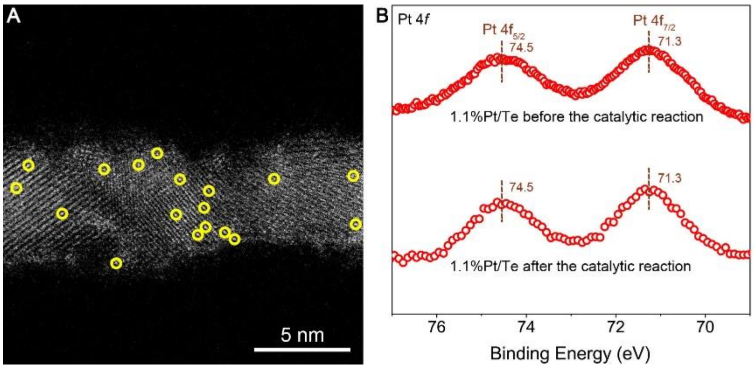

Figure S7. (A) HAADF-STEM image of 1.1%Pt/Te after being recycled for five times. (B) XPS spectra of Pt 4*f* for 1.1%Pt/Te before and after being recycled for five times.

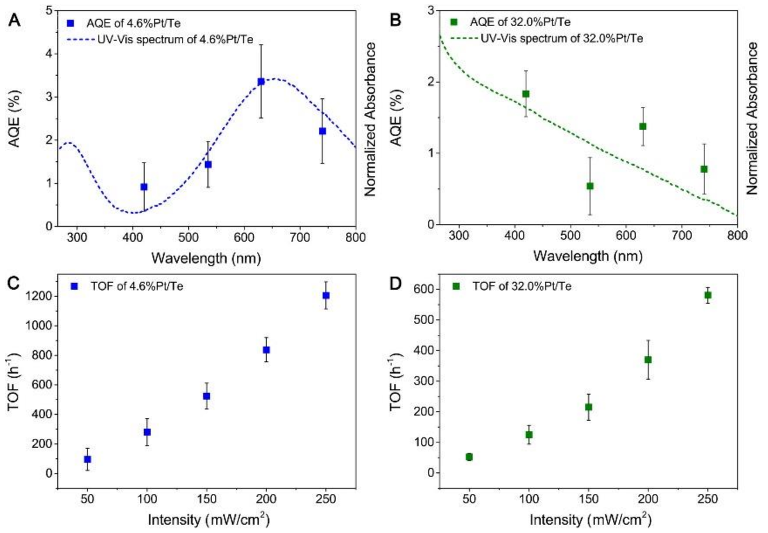

Figure S8. (A, B) Correlation between AQEs of 4.6%Pt/Te and 32.0%Pt/Te with light wavelength. (C, D) Correlation between TOFs of 4.6%Pt/Te and 32.0%Pt/Te with light intensity.

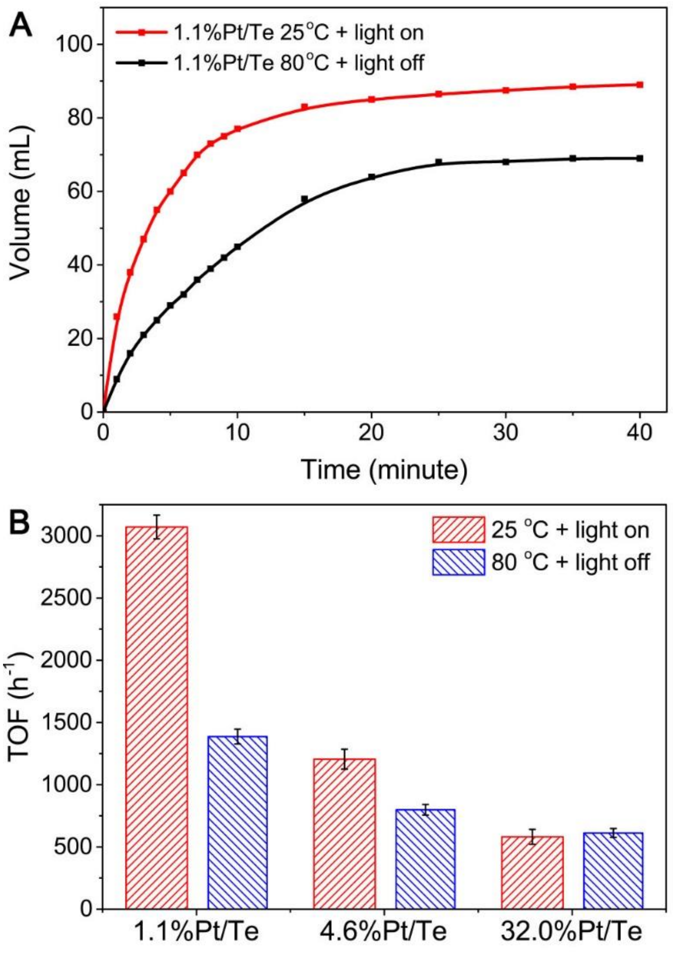

Figure S9. (A) Curves of the reaction of dehydrogenation of HCOOH catalyzed by 1.1%Pt/Te at 25 ^{o}C with light and at 80 ^{o}C without light. (B) Comparison of TOFs for 1.1%Pt/Te, 4.6%Pt/Te, 32.0%Pt/Te at 25 ^{o}C with light and at 80 ^{o}C without light.

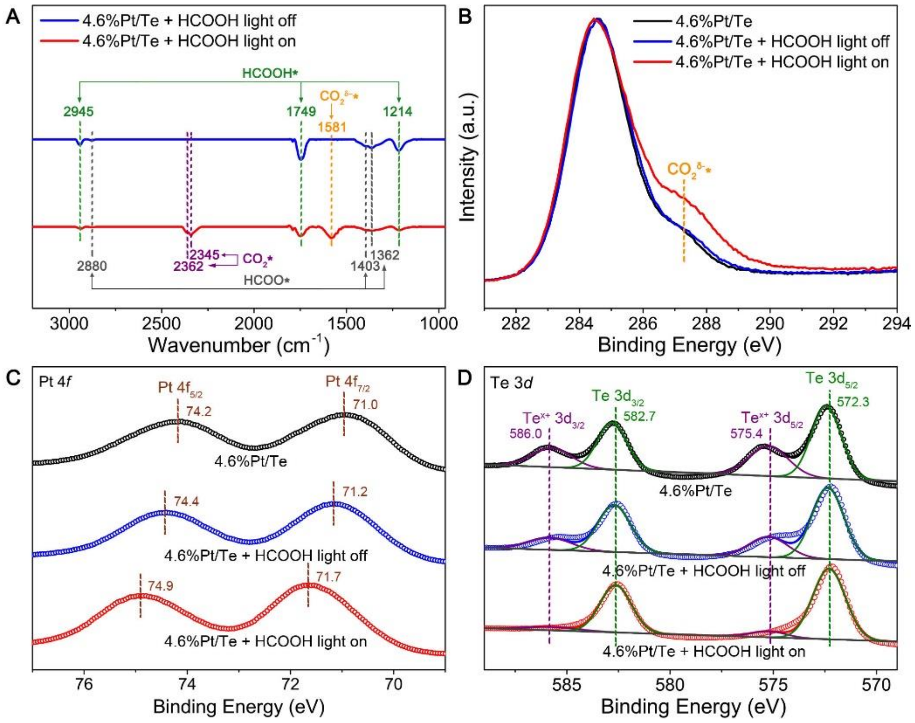

Figure S10. (A) *In situ* DRIFT spectra of 4.6%Pt/Te after the treatment of HCOOH at 25 ^{o}C with/without light for 10 min. (B, C, D) C 1s, Pt 4*f*, and Te 3*d* *quasi in situ* XPS spectra for 4.6%Pt/Te before and after the treatment of HCOOH at 25 ^{o}C with/without light for 10 min.

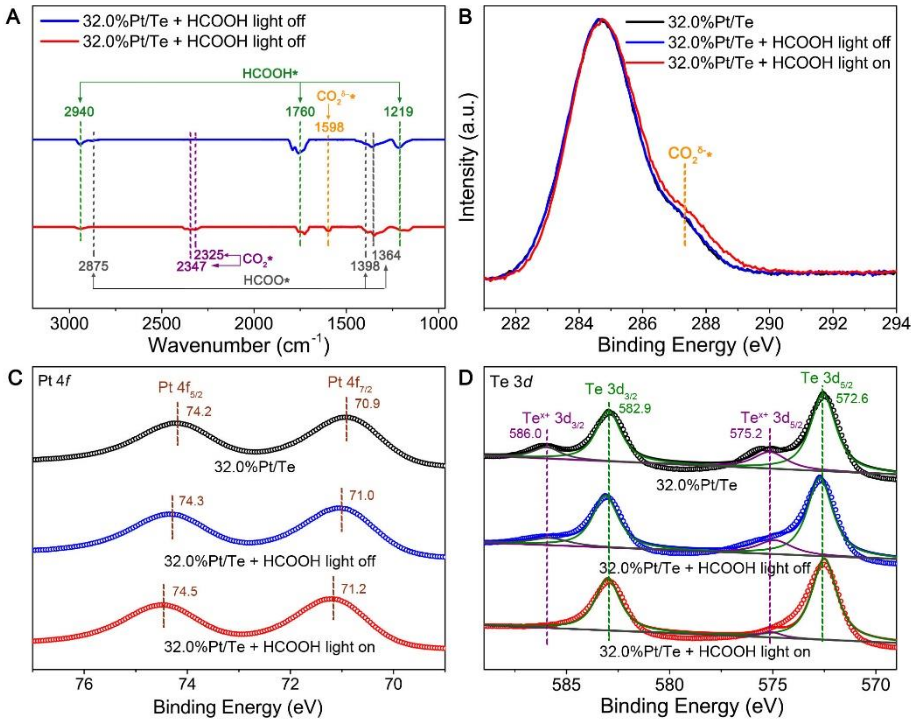

Figure S11. (A) *In situ* DRIFT spectra of 32.0%Pt/Te after the treatment of HCOOH at 25 ^{o}C with/without light for 10 min. (B, C, D) C 1s, Pt 4*f*, and Te 3*d quasi in situ* XPS spectra for 32.0%Pt/Te before and after the treatment of HCOOH at 25 ^{o}C with/without light for 10 min.
